# Exploring the function of protein kinases in schistosomes: perspectives from the laboratory and from comparative genomics

**DOI:** 10.3389/fgene.2014.00229

**Published:** 2014-07-31

**Authors:** Anthony J. Walker, Margarida Ressurreição, Rolf Rothermel

**Affiliations:** Laboratory of Molecular Parasitology, School of Life Sciences, Kingston UniversityKingston upon Thames, UK

**Keywords:** *Schistosoma*, kinome, *Caenorhabditis elegans*, kinase function, cell signaling, schistosomiasis

## Abstract

Eukaryotic protein kinases are well conserved through evolution. The genome of *Schistosoma mansoni*, which causes intestinal schistosomiasis, encodes over 250 putative protein kinases with all of the main eukaryotic groups represented. However, unraveling functional roles for these kinases is a considerable endeavor, particularly as protein kinases regulate multiple and sometimes overlapping cell and tissue functions in organisms. In this article, elucidating protein kinase signal transduction and function in schistosomes is considered from the perspective of the state-of-the-art methodologies used and comparative organismal biology, with a focus on current advances and future directions. Using the free-living nematode *Caenorhabditis elegans* as a comparator we predict roles for various schistosome protein kinases in processes vital for host invasion and successful parasitism such as sensory behavior, growth and development. It is anticipated that the characterization of schistosome protein kinases in the context of parasite function will catalyze cutting edge research into host-parasite interactions and will reveal new targets for developing drug interventions against human schistosomiasis.

## Protein kinases and schistosomes—an overview

Protein kinases are pivotal regulators of cellular function. When activated, these signaling enzymes phosphorylate transcription factors and other intracellular proteins leading to alteration in gene expression and/or other cellular behavior/activities. Elucidating functional roles for protein kinases in humans and other organisms such as *Drosophila melanogaster* and *Caenorhabditis elegans* has sometimes been challenging, particularly because protein kinase pathways can drive multiple functions and influence one another through cross talk (e.g., Krishna and Narang, 2008), the nature of which is often dependent upon “input” signal(s). This presents a conundrum when trying to characterize the functions of protein kinases in organisms that have only relatively recently entered the protein kinase research arena. Schistosomes, which are human blood parasites, arguably fall into this category. The importance of schistosomes, particularly *Schistosoma mansoni, Schistosoma japonicum*, and *Schistosoma haematobium*, is highlighted by the fact that they are responsible for causing the neglected tropical disease human schistosomiasis in over 200 million people worldwide (Steinmann et al., 2006).

The life cycle of these schistosomes is complex (Walker, 2011). Paired adult male and female worms living in the blood vessels of the human definitive host produce eggs that are excreted and hatch in fresh water releasing a miracidium that infects an aquatic intermediate host snail. In the snail the miracidium develops into a mother and then daughter sporocysts that produce cercariae; when released into water these cercariae search out and penetrate the skin of the human host. Each cercaria then transforms into a schistosomule which subsequently enters the circulation and develops into juvenile and then adult worms that pair to stimulate full maturation and egg laying. Considerable morphological and physiological differences exist between these separate schistosome life-cycle stages (Walker, 2011) and thus protein kinase function may be different in each individual life stage, adding a further layer of complexity to role characterization. Also, each life stage will intercept different “input” signals from its environment (Walker, 2011). Such signals will include: light and host-derived surface molecules in the free-living swimming stages (cercariae and miracidia); signaling molecules (e.g., growth factors) in the blood of the endoparasitic stages of both the snail (post-miracidia and mother/daughter sporocyst) and human (schistosomule and adult worm) host; and differences in osmolarity and/or temperature during infection of/release from the host. Furthermore, an immortalized schistosome cell line does not exist meaning that cellular experiments need to be conducted with whole schistosomes, fractions, or lysates thereof. Nevertheless, while challenging, defining roles for kinases in this fascinating parasite will be rewarding, enabling questions concerning cellular regulation, development and homeostasis, particularly during key life-stage transitions to be answered. Such research is also important to develop strategies focusing on drug-mediated kinase modulation in the parasite for therapeutic intervention.

*Schistosoma mansoni, S. japonicum*, and *S. haematobium* genome and transcriptome projects [e.g., (Hu et al., 2003; Verjovski-Almeida et al., 2003; Berriman et al., 2009; The *Schistosoma japonicum* Genome Sequencing and Functional Analysis Consortium, 2009; Protasio et al., 2012; Young et al., 2012)] have provided crucial sequence and expression data to support research into schistosome signaling, and 252 putative protein kinase genes have been found in *S. mansoni* (Andrade et al., 2011). However, the number of protein kinases encoded by these genes remains unknown because of the possibility of alternative splicing. This phenomenon, which increases proteome complexity, likely provides over 900 protein kinases from 445 protein kinase genes in humans, with 209 genes encoding a single kinase (Anamika et al., 2009). Alternative splicing results in potentially diverse functions because protein kinase splice variants can possess different domain architectures (Anamika et al., 2009).

Schistosomes possess putative kinases from all eight main eukaryotic protein kinase groups (Andrade et al., 2011). They also possess upstream receptors and endogenous signaling molecules (Osman et al., 2006; Khayath et al., 2007; Berriman et al., 2009; Oliveira et al., 2009; The *Schistosoma japonicum* Genome Sequencing and Functional Analysis Consortium, 2009; Zamanian et al., 2011; Young et al., 2012). Importantly, in the context of host-parasite interactions, schistosomes have been shown to respond to human insulin (You et al., 2009), transforming growth factor (TGF)-β 1 (Osman et al., 2006), and tumor necrosis factor (TNF)-α (Oliveira et al., 2009), demonstrating that they can bind host signaling molecules and transduce input signals through intact pathways. Here, perspectives on unraveling kinase function in schistosomes are provided that stem from techniques in cell biology developed for other organisms and from comparative genomics using *C. elegans* as a model.

## Schistosome functional kinomics—perspectives from the laboratory

### Studying activated protein kinases in schistosomes

**Figure 1:**
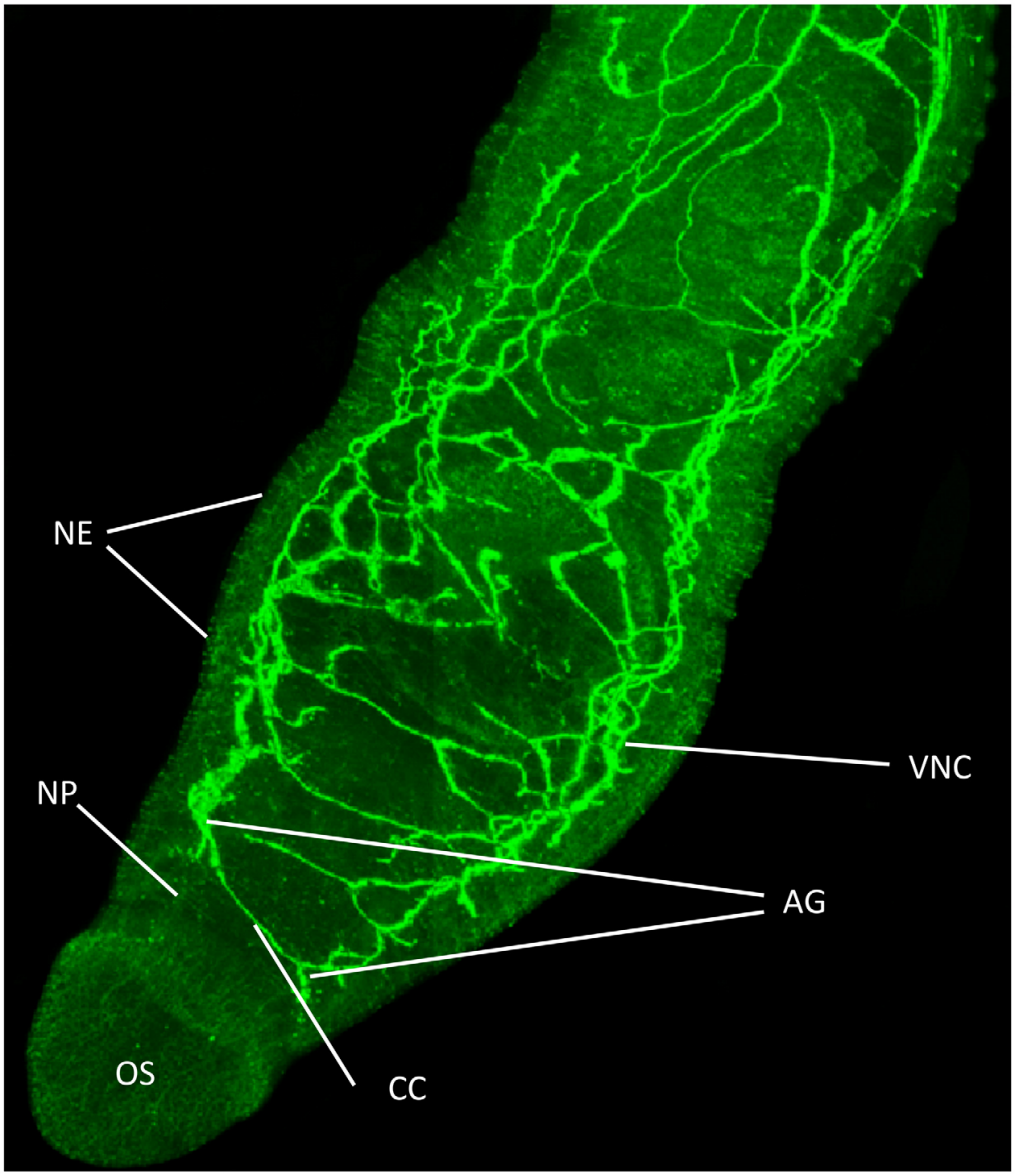
**Functional mapping of protein kinase A (PKA) in intact adult *S. mansoni***. Male worms were fixed immediately after perfusion and stained with anti-phospho antibodies (green) to localize activated PKA. Image shows z-axis projection from intact worm in maximum pixel brightness mode. Intense staining reveals PKA activation particularly associated with the nervous system including in the ventral nerve cords (VNC), connecting cerebral commissures (CC), anterior ganglia (AG), complex nerve plexus (NP) associated with the oral sucker (OS) and nerve endings (NE) at the tegument surface. Bar = 100 μm. Adapted from De Saram et al. (2013).

In our laboratory we have employed “smart” phospho-specific antibodies (Bonetta, 2005) to detect functionally activated protein kinases in life stages of *S. mansoni*. These antibodies, first produced by Cell Signaling Technology (CST; www.cellsignal.com) detect key phosphorylation sites (Tyr, Thr, or Ser) within the kinase that are critical for function. Although such antibodies are generated against phosphorylated human peptide sequences (typically 11–13 amino acids around the phosphorylation site), a number of such sequences are well conserved in kinases of invertebrates allowing certain antibodies to be used after careful validation. For example, anti-phospho antibodies against the mitogen-activated protein kinases (MAPKs) have been used widely in *D. melanogaster* and *C. elegans* (e.g., Gabay et al., 1997; You et al., 2006; Zhuang et al., 2006), and in snails (Plows et al., 2004, 2005) including *B. glabrata*, host to *S. mansoni* (Zahoor et al., 2008). In our approach (Ressurreição et al., 2011a) we first align the predicted *Schistosoma* kinase protein sequence (from www.GeneDB.org) with the orthologous sequence for the respective human kinase and search for conserved site(s) containing the important phosphorylation motif(s). We next select appropriate antibodies (e.g., from CST) and screen them using western blotting with extracts from untreated live parasites, and live parasites treated with an activator for the kinase in question (e.g., anisomycin for p38 MAPK). Increased immunoreactivity of the kinase from the exposed parasite samples provides the first indication of antibody suitability and of functional conservation of the phosphorylated site in schistosomes. Antibodies are then further validated before they are used routinely. This involves experiments such as inhibition assays to block upstream activators of the kinase or block direct kinase activation (Ludtmann et al., 2009), and if possible immunoprecipitation of the phosphorylated kinase followed by kinase assay. In principle, by raising antibodies against phospho-peptides identical to schistosome protein kinase sequences it is possible to produce schistosome-specific anti-phospho antibodies; however, this requires significant investment without knowing whether the phospho site is functionally active. A major benefit of using anti-phospho antibodies is that it is possible to study protein kinase activation with small quantities of protein (e.g., from ~750 schistosomules or one adult worm pair) using western blotting. While for some schistosome protein kinases activity has been determined using activity assays with conserved kinase substrates (Wiest et al., 1992; Swierczewski and Davies, 2009, 2010), the quantity of protein needed is usually considerably greater. A further benefit of phospho-specific antibodies is that they often work in immunohistochemistry allowing the exclusively activated kinase (rather than the just the protein) to be visualized within the intact parasite using fluorescence-based confocal laser scanning microscopy (Figure 1). For this we have coined the term “functional mapping” (De Saram et al., 2013) given that only the activated kinase is detected. This can be valuable to elucidate the apparent distribution of the activated kinase within the parasite enabling hypotheses concerning function to be formulated and subsequently tested (see below). To date we have employed anti-phospho antibodies in *S. mansoni* to explore the kinetics of protein kinase C (PKC) and p38 MAPK activation in miracidia during development to mother sporocysts (Ludtmann et al., 2009; Ressurreição et al., 2011b), to help demonstrate a role for p38 MAPK in miracidia motility (Ressurreição et al., 2011a), and to study protein kinase A (PKA) function in adult worms (De Saram et al., 2013). Our current research using anti-phospho antibodies is focusing further on these pathways and others [e.g., AKT and extracellular signal-regulated kinase (ERK)] in cercariae, schistosomules, and adult worms.

We anticipate that the above tools will also help enable certain protein kinase-mediated signaling pathways to be delineated. Putative pathway maps have been constructed incorporating some schistosome protein kinases including MAPK pathways and others (Wang et al., 2006; Dissous et al., 2007; Berriman et al., 2009; Beckmann et al., 2010b). These are largely predicted using *in silico* data for schistosome pathway components, comparative mechanisms in vertebrates and, in some cases, interaction data from schistosomes obtained using valuable yeast two/three-hybrid screening and immunoprecipitation experiments [e.g., for Src and syk tyrosine kinases and polo-like kinases (Plks) (Quack et al., 2009; Beckmann et al., 2010a,b; Long et al., 2012)]. However, experimental data delineating routes of pathway activation in schistosomes remain negligible and need to be expanded to develop functional pathway maps.

### Deciphering protein kinase function in intact schistosomes

Current strategies for interfering with protein kinases to elucidate function directly within schistosomes focus largely on pharmacological inhibition and conventional RNA interference (RNAi) by double stranded RNA. Probably the most controversial aspect of using kinase inhibitors is their potential to affect “non-target” kinases, particularly at high concentrations (Anastassiadis et al., 2011). However, when functional experiments are performed on intact parasites inhibitor IC50's have little meaning as their values are normally derived from cell-free or single-cell assays. To help mitigate possible non-target effects, careful selection of kinase inhibitors is needed, particularly as they often have different specificities. Sometimes sites of protein-inhibitor interaction have been mapped as illustrated by SB203580 and its target p38 MAPK (Gum et al., 1998; Wang et al., 1998). Such knowledge is valuable as it enables one to ascertain whether the critical residues are conserved in the schistosome kinase, as was found for *S. japonicum* p38 MAPK during our recent work (Ressurreição et al., 2011a). Moreover, performing inhibition experiments with subsequent kinase activity screening (see above) further validates inhibitor use. Experiments with inhibitors/activators have supported roles for kinases in *S. mansoni* including in: (1) miracidia/sporocysts, in which p38 MAPK (Ressurreição et al., 2011b) and PKC (Ludtmann et al., 2009) inhibition restrict and accelerate miracidia-to-mother sporocyst development, respectively, and activation of p38 MAPK attenuates miracidial ciliary motion (Ressurreição et al., 2011a); (2) cercariae and adult worms, in which PKA inhibition kills the parasite (Swierczewski and Davies, 2009, 2010); and (3) adult worms, in which PKA activation stimulates neuromuscular movement (De Saram et al., 2013), insulin and venus kinase receptor inhibition restricts feeding, egg-laying, and results in death (Vanderstraete et al., 2013), and SmTK4 and Plk inhibition suppresses gametogenesis (Beckmann et al., 2010a; Long et al., 2010). Moreover, inhibition and transcriptomic analysis have recently been used to identify a co-operative role for Src kinase and TGFβ in eggshell formation (Buro et al., 2013). By coupling outcomes of pharmacological experiments with *in situ* functional mapping (De Saram et al., 2013)/*in-situ* hybridization (Long et al., 2012), further confidence in terms of identified role can be achieved. Thus, inhibitor-based assays have an important place in functional schistosome kinomic research when used appropriately, and are particularly useful in short-term experiments as RNAi-mediated knockdown of a protein can take several days.

Conventional RNAi has become an invaluable tool for studying protein function in various life stages of *Schistosoma* species (Kalinna and Brindley, 2007; Stefanić et al., 2010; Rinaldi et al., 2011). This approach has been used to silence a large number of schistosome proteins including leucine aminopeptidase, involved in hatching of miracidia (Rinaldi et al., 2009), tetraspanins 1 and 2 that regulate tegument integrity (Tran et al., 2010), cathepsin B, important to schistosome growth (Correnti et al., 2005), and aquaporin, involved in excretion of metabolic waste across the tegument (Faghiri et al., 2010). Challenges with RNAi in schistosomes, however, remain and are considered elsewhere (Stefanić et al., 2010; Dalzell et al., 2012). These include the transient nature of RNAi, variable knockdown between individual parasites, and “knock-down” rather than “knock-out” of gene function, all of which can complicate phenotype analysis. To-date, there are relatively few reports of schistosome protein kinases being targeted by RNAi. These include knockdown of PKA, SmTK4, fibroblast growth factor receptor, TGFβ receptor II and Ca2^+^/calmodulin-dependent protein kinase II, found important for viability, gametogenesis, maintenance of neoblast-like cells, male-female reproductive development, and regulation of praziquantel induced calcium influx in adult worms, respectively (Osman et al., 2006; Swierczewski and Davies, 2009; Beckmann et al., 2010a; Collins et al., 2013; You et al., 2013). Importantly, the interconnected nature of kinase signaling is such that phenotypes caused by RNAi-mediated kinase depletion presumably reflect the aggregate biological consequence of disregulation of several pathways (Sopko and Andrews, 2008). Furthermore, phenotypes may also be masked by increased compensatory expression and subsequent activation of other isotypes and pathways in the face of suppression of any one isotype. Thus, although conventional RNAi is valuable for schistosome kinomics research, interpreting phenotype outcomes can be more challenging than for single gene/single function proteins. Nevertheless, in principle, as proposed for mammals (Moffat and Sabatini, 2006), it should be possible to perform high-throughput RNAi-based screening to delineate schistosome signaling pathways, using downstream phosphorylation events as “readouts” for depletion of “upstream” components such as kinases. Although an immortalized schistosome cell line might appear essential for such experiments, we consider progress could also be achieved using primary cell cultures derived from mechanically or enzymatically dissociated schistosome tissues, and possibly even totipotent stem cells recently identified in this parasite (Collins et al., 2013; Wang et al., 2013).

## Schistosome functional kinomics—perspectives and predictions from comparative genomics

Given that protein kinases have been conserved through evolution, valuable insights into their possible functions in schistosomes can also be gleaned by considering roles for orthologous kinases in organisms in which functional genomics is more advanced. Here, selected *S. mansoni* protein kinases displaying orthology to protein kinases of the well-characterized *C. elegans* have been chosen (Table 1) to illustrate how comparative genomics can help build hypotheses for testing protein kinase function in schistosomes.

**Table 1:**
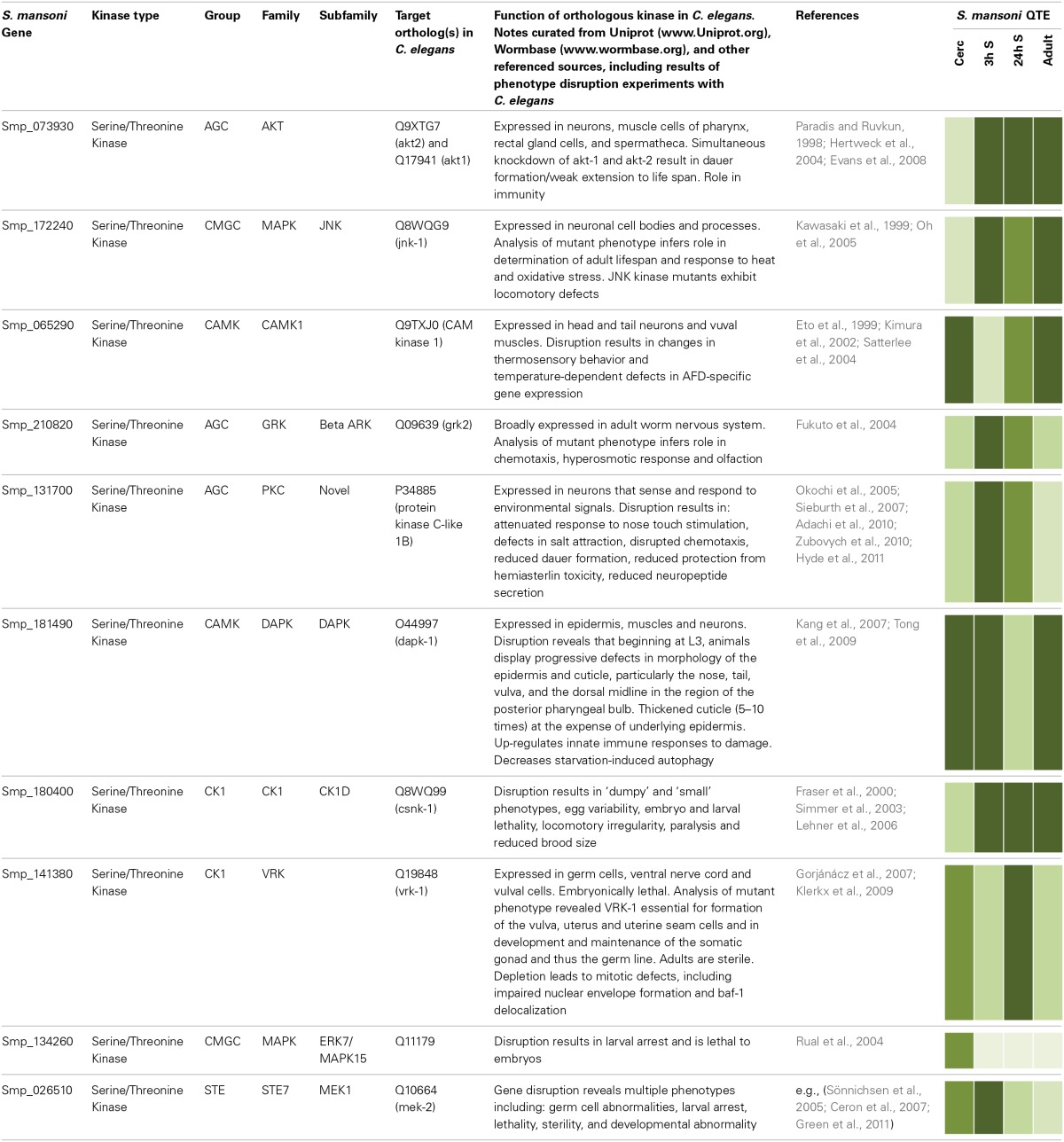
**Selected examples of orthologous protein kinases in *S. mansoni* and *C. elegans* with functional annotations shown for *C. elegans***.

*Relative quantitative transcript expression (QTE) data for cercariae (Cerc), 3 and 24 h schistosomules (3 and 24 h S), and adult worms were extracted from GeneDB [www.Genedb.org (Protasio et al., 2012)]; 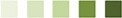 representing 0–0.2, 0.2–0.4, 0.4–0.6, 0.6–0.8, and 0.8–1.0 relative expression, respectively. Smp protein identifiers were queried in PhylomeDB (www.phylomedb.org) to obtain orthologous identifiers for C. elegans proteins, and protein sequence compared using BLAST (http://blast.ncbi.nlm.nih.gov). Curated data on Uniprot (www.Uniprot.org) and Wormbase (www.wormbase.org) were used for phenotype annotation*.

*Caenorhabditis elegans* dauer larvae exhibit distinct morphological and behavioral characteristics in response to environmental duress. AKT-1 and AKT-2, components of the insulin-like pathway, phosphorylate and inhibit the FoxO transcription factor DAF-16 (Paradis and Ruvkun, 1998; Hertweck et al., 2004) regulating dauer formation and lifespan (Hu, 2007), such that DAF-16/FoxO activation in the intestine affects lifespan whereas neuronal activity affects dauer formation (Libina et al., 2003). The orthologous *S. mansoni* Akt gene (Smp_073930) is particularly expressed in the schistosomules and adult worms (Table 1); it is therefore possible to predict that Akt may regulate lifespan in *S. mansoni*. Interestingly, Smp_172240 is orthologous for a c-Jun N-terminal kinase (JNK-1) in *C. elegans*, which acts together with the insulin-like pathway and converges with DAF-16 to further promote lifespan regulation. In fact, *C. elegans* Jnk-1 mutants have reduced lifespan and increased tolerance to heat stress. JNK-1 modulates translocation of DAF-16 to the nucleus where it promotes expression of specific genes to combat environmental stress (Oh et al., 2005). Compared to cercariae, JNK-1 is more highly expressed in 3 h schistosomules (Table 1), which coincides with the considerable environmental change experienced by schistosomes upon entering the warm-blooded definitive host. The presence of *Schistosoma* insulin-like and JNK pathway components, vital to lifespan regulation in *C. elegans*, warrants investigations to decipher whether AKT and JNK regulate growth and development in schistosomes and whether these proteins co-operate in a similar manner. Thus, during our on-going investigations into AKT signaling in schistosomes we shall use the *C. elegans* knowledge base to build and test hypotheses concerning AKT function in different *S. mansoni* life stages.

Smp_065290, orthologous with CAM Kinase-1 in *C. elegans* and responsible for thermosensory behavior (Table 1), has raised expression in cercariae compared to 3 h schistosomules, consistent with cercariae being stimulated by a rising temperature gradients (Cohen et al., 1980). Although *C. elegans* CAMK-1 mutants display thermosensory defects, the mechanisms by which CAMK-1 operates remain to be characterized fully (Satterlee et al., 2004). Disruption of this gene in schistosomes would be interesting to revel whether it mediates host searching/cercarial penetration, possibly together with JNK-1 (orthologous to Smp_172240) that might play a role in thermosensation given its importance to thermotolerence in *C. elegans* (Table 1).

*Schistosoma mansoni* novel PKCε (Smp_131700; Table 1) is orthologous to *C. elegans* novel PKC-1 that plays a role in salt attraction (Adachi et al., 2010). Expression analysis (Table 1) suggests this gene is important in cercariae and early schistosomules. Increasing salinity promotes transformation of cercariae to schistosomules (Samueleson and Stein, 1989), thus, Smp_131700 provides an interesting target for phenotype disruption studies focusing on successful host infection by schistosomes. In *C. elegans*, PKC-1 is involved with ASE-right (ASER) neurons, which sense the Cl ion of NaCl (Adachi et al., 2010). ASER also express the guanylate cyclase receptor, gcy-22, essential in Cl ion sensing, and *C. elegans* gcy-22 mutants show impaired ability of ASER in response to varying salt concentrations (Ortiz et al., 2010). Interestingly, PKC-1 has other roles in *C. elegans* including nose touch stimulation and locomotion with evidence suggesting it acts through the conserved ERK pathway (Hyde et al., 2011). It is thus tempting to predict a further role for *S. mansoni* PKCε in mechanosensation during contact of cercariae to host skin and in schistosome locomotion. Future immunolocalization studies may reveal PKCε in the nervous system of *S. mansoni* as in *C. elegans*.

Other examples illustrated in Table 1 include MAPK15, MEK1, casein kinase 1 (CK1), and vaccinia-related kinase (VRK), disruption of which in *C. elegans* is lethal to larvae, embryos, or both, and a G-protein coupled receptor kinase (GRK) that plays a role in chemotaxis, the hyperosmotic response and olfaction. The death-associated protein kinase (DAPK), expressed at high levels in all stages except 24 h schistosomules, plays an important role in epidermal and cuticle integrity and maintenance in *C. elegans* (Table 1). Given the importance of the schistosome tegument to host immune evasion and parasite survival (Van Hellemond et al., 2006), and the potential for similar effects in schistosomes, studies focusing on this kinase are warranted.

## Schistosome functional kinomics—future perspectives

By integrating various approaches such as those detailed above and benefiting from emerging tools like schistosome transgenesis (Mann et al., 2011), it should be possible to unravel the complexity of protein kinase signaling in schistosomes. Certain protein kinases in schistosomes may ultimately be found to have roles comparable to those in other organisms including *C. elegans*, which can currently be used to generate functional hypotheses to test [see also further lethality predictions (Andrade et al., 2011)]. However, it is worth noting that common functionality may differ particularly as different organisms possess different complements of protein kinases and downstream target proteins, and because tissue expression may differ. To illustrate differences, *in silico* reconstruction of the *S. mansoni* and S. *japonicum* MAPK pathways (Wang et al., 2006; Berriman et al., 2009) shows that while many of the *Schistosoma* cytosolic signaling mechanisms are intact when compared to those of humans, the number of orthologous downstream transcription factors appear fewer, with members such as CREB, p53, c-Myc, and c-Jun strikingly absent. Thus, while hypothetical frameworks for kinase signaling in schistosomes may be built, they do need to be tested.

Schistosome protein kinase research is arguably in its infancy and many questions remain concerning the functional biology of these important enzymes in this parasite. To help address these issues, and to stimulate research on schistosome protein kinases, it is proposed here that key areas for on-going fundamental schistosome kinomic research should include:

- Identification of upstream activators and downstream targets of schistosome protein kinases to enable functional pathway characterization. To include consideration of “input” signals such as host growth factors;
- Functional elucidation of protein kinases beyond the commonly used “gross” phenotypes (e.g., dead/alive, moving/not moving) often evaluated;
- Consideration of all life stages to fully appreciate the complexity of protein kinase signaling in this parasite. This is important because outcomes from one life-stage may offer novel insights into cellular regulation in another life-stage.

By expanding research in the above areas, it should be ultimately possible to integrate fundamental research outcomes and develop a systems level understanding of protein kinase function in schistosomes. In doing so, opportunities will emerge along the way to consider individual kinases as targets for drug-mediated chemotherapy for schistosomiasis. This may include making use of drugs that are available/being considered for therapy of other diseases such as cancer [e.g., (Ali et al., 2009)], modification of such drugs, or development of new multi-species drugs that specifically target *Schistosoma* protein kinases.

### Conflict of interest statement

The authors declare that the research was conducted in the absence of any commercial or financial relationships that could be construed as a potential conflict of interest.

## References

[B1] Adachi T. Kunitomo H. Tomioka M. Ohno H. Okochi Y. Mori I. (2010). Reversal of salt preference is directed by the insulin/PI3K and Gq/PKC signaling in *Caenorhabditis elegans*. Genetics 186, 1309–1319 10.1534/genetics.110.119768 20837997 PMC2998313

[B2] Ali A. S. Ali S. El-Rayes B. F. Philip P. A. Sarkar F. H. (2009). Exploitation of protein kinase C: a useful target for cancer therapy. Cancer Treat. Rev. 35, 1–8 10.1016/j.ctrv.2008.07.006 18778896

[B3] Anamika K. Garnier N. Srinivasan N. (2009). Functional diversity of human protein kinase splice variants marks significant expansion of human kinome. BMC Genomics 10:622 10.1186/1471-2164-10-622 20028505 PMC2805699

[B4] Anastassiadis T. Deacon S. W. Devarajan K. Ma H. Peterson J. R. (2011). Comprehensive assay of kinase catalytic activity reveals features of kinase inhibitor selectivity. Nat. Biotech. 29, 1039–1045 10.1038/nbt.2017 22037377 PMC3230241

[B5] Andrade L. F. Nahum L. A. Avelar L. G. A. Silva L. L. Zerlotini A. Ruiz J. C. (2011). Eukaryotic protein kinases (ePKs) of the helminth parasite *Schistosoma mansoni*. BMC Genomics 12:215 10.1186/1471-2164-12-215 21548963 PMC3117856

[B6] Beckmann S. Buro C. Dissous C. Hirzmann J. Grevelding C. G. (2010a). The Syk kinase SmTK4 of *Schistosoma mansoni* is involved in the regulation of spermatogenesis and oogenesis. PLoS Pathog. 6:e1000769 10.1371/journal.ppat.1000769 20169182 PMC2820527

[B7] Beckmann S. Quack T. Burmeister C. Buro C. Long T. Dissous C. (2010b). *Schistosoma mansoni*: signal transduction processes during the development of the reproductive organs. Parasitology 137, 497–520 10.1017/S0031182010000053 20163751

[B8] Berriman M. Haas B. J. LoVerde P. T. Wilson R. A. Dillon G. P. Cerqueira G. C. (2009). The genome of the blood fluke *Schistosoma mansoni*. Nature 460, 352–358 10.1038/nature08160 19606141 PMC2756445

[B9] Bonetta L. (2005). Probing the kinome. Nat. Methods 2, 225–232 10.1038/nmeth0305-225

[B10] Buro C. Oliveira K. C. Lu Z. Leutner S. Beckmann S. Dissous C. (2013). Transcriptome analyses of inhibitor-treated schistosome females provide evidence for cooperating Src-kinase and TGFβ receptor pathways controlling mitosis and eggshell formation. PLoS Pathog. 9:e1003448 10.1371/journal.ppat.1003448 23785292 PMC3681755

[B11] Ceron J. Rual J.-F. Chandra A. Dupuy D. Vidal M. van den Heuvel S. (2007). Large-scale RNAi screens identify novel genes that interact with the *C. elegans* retinoblastoma pathway as well as splicing-related components with synMuv B activity. BMC Dev. Biol. 7:30 10.1186/1471-213X-7-30 17417969 PMC1863419

[B12] Cohen L. Neimark H. Eveland L. (1980). *Schistosoma mansoni*: response of cercariae to a thermal gradient. J. Parasitol. 66, 362–364 7391881

[B13] Collins J. J. Wang B. Lambrus B. G. Tharp M. E. Iyer H. Newmark P. A. (2013). Adult somatic stem cells in the human parasite *Schistosoma mansoni*. Nature 494, 476–479 10.1038/nature11924 23426263 PMC3586782

[B14] Correnti J. M. Brindley P. J. Pearce E. J. (2005). Long-term suppression of cathepsin B levels by RNA interference retards schistosome growth. Mol. Biochem. Parasitol. 143, 209–215 10.1016/j.molbiopara.2005.06.007 16076506

[B15] Dalzell J. J. Warnock N. D. McVeigh P. Marks N. J. Mousley A. Atkinson L. (2012). Considering RNAi experimental design in parasitic helminths. Parasitology 139, 589–604 10.1017/S0031182011001946 22216952

[B16] De Saram P. S. R. Ressurreição M. Davies A. J. Rollinson D. Emery A. M. Walker A. J. (2013). Functional mapping of protein kinase A reveals its importance in adult *Schistosoma mansoni* motor activity. PLoS Negl. Trop. Dis. 7:e1988 10.1371/journal.pntd.0001988 23326613 PMC3542114

[B17] Dissous C. Ahier A. Khayath N. (2007). Protein tyrosine kinases as new potential targets against human schistosomiasis. BioEssays 29, 1281–1288 10.1002/bies.20662 18022808

[B18] Eto K. Takahashi N. Kimura Y. Masuho Y. Arai K. Muramatsu M. A. (1999). Ca(2+)/Calmodulin-dependent protein kinase cascade in *Caenorhabditis elegans*. Implication in transcriptional activation. J. Biol. Chem. 274, 22556–22562 10.1074/jbc.274.32.22556 10428833

[B19] Evans E. A. Chen W. C. Tan M.-W. (2008). The DAF-2 insulin-like signaling pathway independently regulates aging and immunity in C. elegans. Aging Cell 7, 879–893 10.1111/j.1474-9726.2008.00435.x 18782349 PMC2630471

[B20] Faghiri Z. Camargo S. M. R. Huggel K. Forster I. C. Ndegwa D. Verrey F. (2010). The tegument of the human parasitic worm *Schistosoma mansoni* as an excretory organ: the surface aquaporin SmAQP is a lactate transporter. PLoS ONE 5:e10451 10.1371/journal.pone.0010451 20454673 PMC2862721

[B21] Fraser A. G. Kamath R. S. Zipperlen P. Martinez-Campos M. Sohrmann M. Ahringer J. (2000). Functional genomic analysis of *C. elegans* chromosome I by systematic RNA interference. Nature 408, 325–330 10.1038/35042517 11099033

[B22] Fukuto H. S. Ferkey D. M. Apicella A. J. Lans H. Sharmeen T. Chen W. (2004). G protein-coupled receptor kinase function is essential for chemosensation in C. elegans. Neuron 42, 581–593 10.1016/S0896-6273(04)00252-1 15157420

[B23] Gabay L. Seger R. Shilo B. Z. (1997). MAP kinase *in situ* activation atlas during *Drosophila* embryogenesis. Development 124, 3535–3541 9342046 10.1242/dev.124.18.3535

[B24] Gorjánácz M. Klerkx E. P. F. Galy V. Santarella R. López-Iglesias C. Askjaer P. (2007). *Caenorhabditis elegans* BAF-1 and its kinase VRK-1 participate directly in post-mitotic nuclear envelope assembly. EMBO J. 26, 132–143 10.1038/sj.emboj.7601470 17170708 PMC1782363

[B25] Green R. A. Kao H.-L. Audhya A. Arur S. Mayers J. R. Fridolfsson H. N. (2011). A high-resolution *C. elegans* essential gene network based on phenotypic profiling of a complex tissue. Cell 145, 470–482 10.1016/j.cell.2011.03.037 21529718 PMC3086541

[B26] Gum R. J. McLaughlin M. M. Kumar S. Wang Z. Bower M. J. Lee J. C. (1998). Acquisition of sensitivity of stress-activated protein kinases to the p38 inhibitor, SB 203580, by alteration of one or more amino acids within the ATP binding pocket. J. Biol. Chem. 273, 15605–15610 10.1074/jbc.273.25.15605 9624152

[B27] Hertweck M. Göbel C. Baumeister R. (2004). *C. elegans* SGK-1 is the critical component in the Akt/PKB kinase complex to control stress response and life span. Dev. Cell 6, 577–588 10.1016/S1534-5807(04)00095-4 15068796

[B28] Hu P. J. (2007). Dauer. WormBook 1–19 10.1895/wormbook.1.144.1 17988074 PMC2890228

[B29] Hu W. Yan Q. Shen D.-K. Liu F. Zhu Z.-D. Song H.-D. (2003). Evolutionary and biomedical implications of a *Schistosoma japonicum* complementary DNA resource. Nat. Genet. 35, 139–147 10.1038/ng1236 12973349

[B30] Hyde R. Corkins M. E. Somers G. A. Hart A. C. (2011). PKC-1 acts with the ERK MAPK signaling pathway to regulate *Caenorhabditis* elegans mechanosensory response. Genes Brain Behav. 10, 286–298 10.1111/j.1601-183X.2010.00667.x 21143768 PMC3664539

[B31] Kalinna B. H. Brindley P. J. (2007). Manipulating the manipulators: advances in parasitic helminth transgenesis and RNAi. Trends Parasitol. 23, 197–204 10.1016/j.pt.2007.03.007 17383233

[B32] Kang C. You Y. Avery L. (2007). Dual roles of autophagy in the survival of *Caenorhabditis elegans* during starvation. Genes Dev. 21, 2161–2171 10.1101/gad.1573107 17785524 PMC1950855

[B33] Kawasaki M. Hisamoto N. Iino Y. Yamamoto M. Ninomiya-Tsuji J. Matsumoto K. (1999). A *Caenorhabditis elegans* JNK signal transduction pathway regulates coordinated movement via type-D GABAergic motor neurons. EMBO J. 18, 3604–3615 10.1093/emboj/18.13.3604 10393177 PMC1171439

[B34] Khayath N. Vicogne J. Ahier A. BenYounes A. Konrad C. Trolet J. (2007). Diversification of the insulin receptor family in the helminth parasite *Schistosoma mansoni*. FEBS J. 274, 659–676 10.1111/j.1742-4658.2006.05610.x 17181541

[B35] Kimura Y. Corcoran E. E. Eto K. Gengyo-Ando K. Muramatsu M.-A. Kobayashi R. (2002). A CaMK cascade activates CRE-mediated transcription in neurons of *Caenorhabditis elegans*. EMBO Rep. 3, 962–966 10.1093/embo-reports/kvf191 12231504 PMC1307624

[B36] Klerkx E. P. F. Alarcón P. Waters K. Reinke V. Sternberg P. W. Askjaer P. (2009). Protein kinase VRK-1 regulates cell invasion and EGL-17/FGF signaling in *Caenorhabditis elegans*. Dev. Biol. 335, 12–21 10.1016/j.ydbio.2009.08.007 19679119 PMC3378332

[B37] Krishna M. Narang H. (2008). The complexity of mitogen-activated protein kinases (MAPKs) made simple. Cell. Mol. Life Sci. 65, 3525–3544 10.1007/s00018-008-8170-7 18668205 PMC11131782

[B38] Lehner B. Calixto A. Crombie C. Tischler J. Fortunato A. Chalfie M. (2006). Loss of LIN-35, the *Caenorhabditis elegans* ortholog of the tumor suppressor p105Rb, results in enhanced RNA interference. Genome Biol. 7:R4 10.1186/gb-2006-7-1-r4 16507136 PMC1431716

[B39] Libina N. Berman J. R. Kenyon C. (2003). Tissue-specific activities of *C. elegans* DAF-16 in the regulation of lifespan. Cell 115, 489–502 10.1016/S0092-8674(03)00889-4 14622602

[B40] Long T. Cailliau K. Beckmann S. Browaeys E. Trolet J. Grevelding C. G. (2010). *Schistosoma mansoni* polo-like kinase 1: a mitotic kinase with key functions in parasite reproduction. Int. J. Parasitol. 40, 1075–1086 10.1016/j.ijpara.2010.03.002 20350550

[B41] Long T. Vanderstraete M. Cailliau K. Morel M. Lescuyer A. Gouignard N. (2012). SmSak, the second polo-like kinase of the helminth parasite *Schistosoma mansoni*: conserved and unexpected roles in meiosis. PLoS ONE 7:e40045 10.1371/journal.pone.0040045 22768216 PMC3386946

[B42] Ludtmann M. H. R. Rollinson D. Emery A. M. Walker A. J. (2009). Protein kinase C signalling during miracidium to mother sporocyst development in the helminth parasite, *Schistosoma mansoni*. Int. J. Parasitol. 39, 1223–1233 10.1016/j.ijpara.2009.04.002 19394337

[B43] Mann V. H. Suttiprapa S. Rinaldi G. Brindley P. J. (2011). Establishing transgenic schistosomes. PLoS Negl. Trop. Dis. 5:e1230 10.1371/journal.pntd.0001230 21912709 PMC3166048

[B44] Moffat J. Sabatini D. M. (2006). Building mammalian signalling pathways with RNAi screens. Nat. Rev. Mol. Cell Biol. 7, 177–187 10.1038/nrm1860 16496020

[B45] Oh S. W. Mukhopadhyay A. Svrzikapa N. Jiang F. Davis R. J. Tissenbaum H. A. (2005). JNK regulates lifespan in *Caenorhabditis elegans* by modulating nuclear translocation of forkhead transcription factor/DAF-16. Proc. Natl. Acad. Sci. U.S.A. 102, 4494–4499 10.1073/pnas.0500749102 15767565 PMC555525

[B46] Okochi Y. Kimura K. D. Ohta A. Mori I. (2005). Diverse regulation of sensory signaling by *C. elegans* nPKC-epsilon/eta TTX-4. EMBO J. 24, 2127–2137 10.1038/sj.emboj.7600697 15920475 PMC1150891

[B47] Oliveira K. C. Carvalho M. L. P. Venancio T. M. Miyasato P. A. Kawano T. DeMarco R. (2009). Identification of the *Schistosoma mansoni* TNF-alpha receptor gene and the effect of human TNF-alpha on the parasite gene expression profile. PLoS Negl. Trop. Dis. 3:e556 10.1371/journal.pntd.0000556 19956564 PMC2779652

[B48] Ortiz C. O. Etchberger J. F. Posy S. L. Frøkjaer-Jensen C. Lockery S. Honig B. (2006). Searching for neuronal left/right asymmetry: genomewide analysis of nematode receptor-type guanylyl cyclases. Genetics 173, 131–149 10.1534/genetics.106.055749 16547101 PMC1461427

[B49] Osman A. Niles E. G. Verjovski-Almeida S. LoVerde P. T. (2006). *Schistosoma mansoni* TGF-beta receptor II: role in host ligand-induced regulation of a schistosome target gene. PLoS Pathog. 2:e54 10.1371/journal.ppat.0020054 16789838 PMC1479047

[B50] Paradis S. Ruvkun G. (1998). *Caenorhabditis elegans* Akt/PKB transduces insulin receptor-like signals from AGE-1 PI3 kinase to the DAF-16 transcription factor. Genes Dev. 12, 2488–2498 10.1101/gad.12.16.2488 9716402 PMC317081

[B51] Plows L. D. Cook R. T. Davies A. J. Walker A. J. (2004). Activation of extracellular-signal regulated kinase is required for phagocytosis by *Lymnaea stagnalis* haemocytes. Biochim. Biophys. Acta 1692, 25–33 10.1016/j.bbamcr.2004.03.002 15158361

[B52] Plows L. D. Cook R. T. Davies A. J. Walker A. J. (2005). Carbohydrates that mimic schistosome surface coat components affect ERK and PKC signalling in *Lymnaea stagnalis* haemocytes. Int. J. Parasitol. 35, 293–302 10.1016/j.ijpara.2004.11.012 15722081

[B53] Protasio A. V. Tsai I. J. Babbage A. Nichol S. Hunt M. Aslett M. A. (2012). A systematically improved high quality genome and transcriptome of the human blood fluke *Schistosoma mansoni*. PLoS Negl. Trop. Dis. 6:e1455 10.1371/journal.pntd.0001455 22253936 PMC3254664

[B54] Quack T. Knobloch J. Beckmann S. Vicogne J. Dissous C. Grevelding C. G. (2009). The formin-homology protein SmDia interacts with the Src kinase SmTK and the GTPase SmRho1 in the gonads of *Schistosoma mansoni*. PLoS ONE 4:e6998 10.1371/journal.pone.0006998 19746159 PMC2734992

[B55] Ressurreição M. Rollinson D. Emery A. M. Walker A. J. (2011a). A role for p38 MAPK in the regulation of ciliary motion in a eukaryote. BMC Cell Biol. 12:6 10.1186/1471-2121-12-6 21269498 PMC3040701

[B56] Ressurreição M. Rollinson D. Emery A. M. Walker A. J. (2011b). A role for p38 mitogen-activated protein kinase in early post-embryonic development of *Schistosoma mansoni*. Mol. Biochem. Parasitol. 180, 51–55 10.1016/j.molbiopara.2011.07.002 21787807

[B57] Rinaldi G. Morales M. E. Alrefaei Y. N. Cancela M. Castillo E. Dalton J. P. (2009). RNA interference targeting leucine aminopeptidase blocks hatching of *Schistosoma mansoni* eggs. Mol. Biochem. Parasitol. 167, 118–126 10.1016/j.molbiopara.2009.05.002 19463860 PMC2705689

[B58] Rinaldi G. Okatcha T. I. Popratiloff A. Ayuk M. A. Suttiprapa S. Mann V. H. (2011). Genetic manipulation of *Schistosoma haematobium*, the neglected schistosome. PLoS Negl. Trop. Dis. 5:e1348 10.1371/journal.pntd.0001348 22022628 PMC3191139

[B59] Rual J.-F. Ceron J. Koreth J. Hao T. Nicot A.-S. Hirozane-Kishikawa T. (2004). Toward improving *Caenorhabditis elegans* phenome mapping with an ORFeome-based RNAi library. Genome Res. 14, 2162–2168 10.1101/gr.2505604 15489339 PMC528933

[B60] Samueleson J. C. Stein L. D. (1989). *Schistosoma mansoni*: increasing saline concentration signals cercariae to transform to schistosomula. Exp. Parasitol. 69, 23–29 2731585 10.1016/0014-4894(89)90167-7

[B61] Satterlee J. S. Ryu W. S. Sengupta P. (2004). The CMK-1 CaMKI and the TAX-4 Cyclic nucleotide-gated channel regulate thermosensory neuron gene expression and function in C. elegans. Curr. Biol. 14, 62–68 10.1016/j.cub.2003.12.030 14711416

[B62] Sieburth D. Madison J. M. Kaplan J. M. (2007). PKC-1 regulates secretion of neuropeptides. Nat. Neurosci. 10, 49–57 10.1038/nn1810 17128266

[B63] Simmer F. Moorman C. van der Linden A. M. Kuijk E. van den Berghe P. V. E. Kamath R. S. (2003). Genome-wide RNAi of *C. elegans* using the hypersensitive rrf-3 strain reveals novel gene functions. PLoS Biol. 1:e12 10.1371/journal.pbio.0000012 14551910 PMC212692

[B64] Sönnichsen B. Koski L. B. Walsh A. Marschall P. Neumann B. Brehm M. (2005). Full-genome RNAi profiling of early embryogenesis in *Caenorhabditis elegans*. Nature 434, 462–469 10.1038/nature03353 15791247

[B65] Sopko R. Andrews B. J. (2008). Linking the kinome and phosphorylome–a comprehensive review of approaches to find kinase targets. Mol. Biosyst. 4, 920–933 10.1039/b801724g 18704230

[B66] Stefanić S. Dvořák J. Horn M. Braschi S. Sojka D. Ruelas D. S. (2010). RNA interference in *Schistosoma mansoni* schistosomula: selectivity, sensitivity and operation for larger-scale screening. PLoS Negl. Trop. Dis. 4:e850 10.1371/journal.pntd.0000850 20976050 PMC2957409

[B67] Steinmann P. Keiser J. Bos R. Tanner M. Utzinger J. (2006). Schistosomiasis and water resources development: systematic review, meta-analysis, and estimates of people at risk. Lancet Infect. Dis. 6, 411–425 10.1016/S1473-3099(06)70521-7 16790382

[B68] Swierczewski B. E. Davies S. J. (2009). A schistosome cAMP-dependent protein kinase catalytic subunit is essential for parasite viability. PLoS Negl. Trop. Dis. 3:e505 10.1371/journal.pntd.0000505 19707280 PMC2724707

[B69] Swierczewski B. E. Davies S. J. (2010). Developmental regulation of protein kinase A expression and activity in *Schistosoma mansoni*. Int. J. Parasitol. 40, 929–935 10.1016/j.ijpara.2010.01.001 20097200 PMC2875359

[B70] The *Schistosoma japonicum* Genome Sequencing Functional Analysis Consortium (2009). The *Schistosoma japonicum* genome reveals features of host-parasite interplay. Nature 460, 345–351 10.1038/nature08140 19606140 PMC3747554

[B71] Tong A. Lynn G. Ngo V. Wong D. Moseley S. L. Ewbank J. J. (2009). Negative regulation of *Caenorhabditis elegans* epidermal damage responses by death-associated protein kinase. Proc. Natl. Acad. Sci. U.S.A. 106, 1457–1461 10.1073/pnas.0809339106 19164535 PMC2629440

[B72] Tran M. H. Freitas T. C. Cooper L. Gaze S. Gatton M. L. Jones M. K. (2010). Suppression of mRNAs encoding tegument tetraspanins from *Schistosoma mansoni* results in impaired tegument turnover. PLoS Pathog. 6:e1000840 10.1371/journal.ppat.1000840 20419145 PMC2855321

[B73] Vanderstraete M. Gouignard N. Cailliau K. Morel M. Lancelot J. Bodart J.-F. (2013). Dual targeting of insulin and venus kinase receptors of *Schistosoma mansoni* for novel anti-schistosome therapy. PLoS Negl. Trop. Dis. 7:e2226 10.1371/journal.pntd.0002226 23696913 PMC3656120

[B74] Van Hellemond J. J. Retra K. Brouwers J. F. H. M. van Balkom B. W. M. Yazdanbakhsh M. Shoemaker C. B. (2006). Functions of the tegument of schistosomes: clues from the proteome and lipidome. Int. J. Parasitol. 36, 691–699 10.1016/j.ijpara.2006.01.007 16545817

[B75] Verjovski-Almeida S. DeMarco R. Martins E. A. L. Guimarães P. E. M. Ojopi E. P. B. Paquola A. C. M. (2003). Transcriptome analysis of the acoelomate human parasite *Schistosoma mansoni*. Nat. Genet. 35, 148–157 10.1038/ng1237 12973350

[B76] Walker A. J. (2011). Insights into the functional biology of schistosomes. Parasit. Vectors 4:203 10.1186/1756-3305-4-203 22013990 PMC3206467

[B77] Wang B. Collins J. J. Newmark P. A. (2013). Functional genomic characterization of neoblast-like stem cells in larval *Schistosoma mansoni*. Elife 2:e00768 10.7554/eLife.00768 23908765 PMC3728622

[B78] Wang L. Yang Z. Li Y. Yu F. Brindley P. J. McManus D. P. (2006). Reconstruction and *in silico* analysis of the MAPK signaling pathways in the human blood fluke, *Schistosoma japonicum*. FEBS Lett. 580, 3677–3686 10.1016/j.febslet.2006.05.055 16765950

[B79] Wang Z. Canagarajah B. J. Boehm J. C. Kassisà S. Cobb M. H. Young P. R. (1998). Structural basis of inhibitor selectivity in MAP kinases. Structure 6, 1117–1128 9753691 10.1016/s0969-2126(98)00113-0

[B80] Wiest P. M. Burnham D. C. Olds G. R. Bowen W. D. (1992). Developmental expression of protein kinase C activity in *Schistosoma mansoni*. Am. J. Trop. Med. Hyg. 46, 358–365 1558275 10.4269/ajtmh.1992.46.358

[B81] You H. McManus D. P. Hu W. Smout M. J. Brindley P. J. Gobert G. N. (2013). Transcriptional responses of *in vivo* praziquantel exposure in schistosomes identifies a functional role for calcium signalling pathway member CamKII. PLoS Pathog. 9:e1003254 10.1371/journal.ppat.1003254 23555262 PMC3610926

[B82] You H. Zhang W. Moertel L. McManus D. P. Gobert G. N. (2009). Transcriptional profiles of adult male and female *Schistosoma japonicum* in response to insulin reveal increased expression of genes involved in growth and development. Int. J. Parasitol. 39, 1551–1559 10.1016/j.ijpara.2009.06.006 19596015

[B83] You Y. Kim J. Cobb M. Avery L. (2006). Starvation activates MAP kinase through the muscarinic acetylcholine pathway in *Caenorhabditis elegans* pharynx. Cell Metab. 3, 237–245 10.1016/j.cmet.2006.02.012 16581001 PMC3433278

[B84] Young N. D. Jex A. R. Li B. Liu S. Yang L. Xiong Z. (2012). Whole-genome sequence of *Schistosoma haematobium*. Nat. Genet. 44, 221–225 10.1038/ng.1065 22246508

[B85] Zahoor Z. Davies A. J. Kirk R. S. Rollinson D. Walker A. J. (2008). Disruption of ERK signalling in *Biomphalaria glabrata* defence cells by *Schistosoma mansoni*: implications for parasite survival in the snail host. Dev. Comp. Immunol. 32, 1561–1571 10.1016/j.dci.2008.05.014 18619674

[B86] Zamanian M. Kimber M. J. McVeigh P. Carlson S. A. Maule A. G. Day T. A. (2011). The repertoire of G protein-coupled receptors in the human parasite *Schistosoma mansoni* and the model organism *Schmidtea mediterranea*. BMC Genomics 12:596 10.1186/1471-2164-12-596 22145649 PMC3261222

[B87] Zhuang Z.-H. Zhou Y. Yu M.-C. Silverman N. Ge B.-X. (2006). Regulation of *Drosophila* p38 activation by specific MAP2 kinase and MAP3 kinase in response to different stimuli. Cell. Signal. 18, 441–448 10.1016/j.cellsig.2005.05.013 16014325

[B88] Zubovych I. O. Straud S. Roth M. G. (2010). Mitochondrial dysfunction confers resistance to multiple drugs in *Caenorhabditis elegans*. Mol. Biol. Cell 21, 956–969 10.1091/mbc.E09-08-0673 20089839 PMC2836976

